# The neuroprotective effect of human primary astrocytes in multiple sclerosis: *In vitro* model

**DOI:** 10.1371/journal.pone.0300203

**Published:** 2024-04-02

**Authors:** Amer Imraish, Tuqa Abu Thiab, Mohammad Alsalem, Saeed Dahbour, Hiba khleif, Basha’er Abu-Irmaileh, Raneen Qasem, Khalid El-Salem

**Affiliations:** 1 Department of Biological Sciences, School of Science, The University of Jordan, Amman, Jordan; 2 Department of Anatomy and Histology, School of Medicine, The University of Jordan, Amman, Jordan; 3 Department of Neurology, Jordan University Hospital, The University of Jordan, Amman, Jordan; 4 Hamdi Mango Centre for Scientific Research, The University of Jordan, Amman, Jordan; 5 Faculty of Medicine, Jordan University of Science and Technology, Irbid, Jordan; University of Louisville, UNITED STATES

## Abstract

Recent studies highlighted the role of astrocytes in neuroinflammatory diseases, particularly multiple sclerosis, interacting closely with other CNS components but also with the immune cells. However, due to the difficulty in obtaining human astrocytes, their role in these pathologies is still unclear. In this study we develop an astrocyte *in vitro* model to evaluate their role in multiple sclerosis after being treated with CSF isolated from both healthy and MS diagnosed patients. Gene expression and ELISA assays reveal that several pro-inflammatory markers IL-1β, TNF-α and IL-6, were significantly downregulated in astrocytes treated with MS-CSF. In contrast, neurotrophic survival, and growth factors, and GFAP, BDNF, GDNF and VEGF, were markedly elevated upon the same treatment. In summary, this study supports the notion of the astrocyte involvement in MS. The results reveal the neuroprotective role of astrocyte in MS pathogenicity by suppressing excessive inflammation and increasing the expression of tropic factors.

## Introduction

Multiple sclerosis (MS) is a neurodegenerative autoimmune disease of the central nervous system (CNS). Inflammation, demyelination, axon damage, and ultimately axon loss are its hallmarks [1,2]. The etiology of MS is still unknown, but the early stages of the disease are initiated in the periphery where the access of blood immune cells into the CNS is restricted. This is achieved by the endothelial cells in the blood brain barrier (BBB) with its tight junctions and specialized transporters known as adhesion molecules [3]. Once activated in the periphery, T cells and B cells, together with macrophages infiltrate into the CNS. There, the afore-mentioned autoreactive cells start a cascade of activation of cells in the CNS. A variety of cytokines are secreted by inflammatory infiltrated cells, including interleukins (ILs), tumor necrosis factor α (TNF-α), interferons (IFNs), and transforming growth factor β (TGF-β) [4–6]. Some of these cytokines and other cytotoxic molecules trigger CNS cells and activate signaling pathways that lead to degenerative cascade that includes oxidative stress followed by axonal demyelination [3].

Within the CNS, astrocytes are considered the main residents and the most abundant glial cells there [7]. Their branching processes form a meshwork that spans regions of the brain. Astrocytes are essential in supporting neuronal functions as well as in maintaining brain homeostasis through synaptogenesis regulation, gliotransmitters release, and extracellular matrix formation [8–11]. They also form the glia limitans around blood vessels, contributing to the blood-brain barrier (BBB) that restricts immune cell access to the CNS [7]. Astrocytes are defined by their expression of glial fibrillary acidic protein (GFAP) and are also a source of vascular endothelial growth factor (VEGF), a factor that participates in the development of the BBB endothelial cells but when overexpressed, VEGF leads to oxidative stress in these cells [11].

In MS, astrocytes are activated by various mediators secreted by infiltrating immune cells and damaged CNS cells [9,11]. Due to their direct contact with the BBB, astrocytes are the first cells to be activated by infiltrating cells in the CNS [12]. Increased GFAP expression and the production of pro-inflammatory and anti-inflammatory cytokines including TNF-α, IL-β, IL-6, IL-8, IL-10 and growth factors, such as brain-derived growth factor (BDNF) and glial cell line-derived neurotrophic factor (GDNF), are caused by this activation [13,14]. Besides, astrocytes activation enhances the expression of genes relevant to oxidative stress, BBB breakdown, and inflammation [11,15]. On the other hand, astrocytes are key players in neuronal repair mechanisms during inflammatory diseases. In response to CNS injury, astrocytes form a glial scar, promoting factors that enhance neuronal repair and remyelination [15]. Among their neuroprotective functions, astrocytes also promote precursor cells differentiation into oligodendrocytes, the a condition that favours remyelination [12].

Demyelination during MS results in the leakage of certain molecules, such as neurofilaments, into the extracellular fluid and consequently into the cerebrospinal fluid (CSF). Furthermore, since the CSF and the interstitial fluid of the nervous system are adjoining, infiltrating inflammatory cells release their secretome into the CSF [16,17]. Thus, CSF composition reflects the micro-environment of the CNS [18,19]. Typical CSF analysis in MS includes elevated count of leukocytes, an indication of CNS inflammatory events.

In this study, CSF samples from healthy individuals and MS patients were collected to investigate their effects on cultured astrocytes. The aim is to understand how astrocytes respond to the secretome present in CSF and whether they play a protective role in MS or exacerbate the disease symptoms. Growth factors and inflammatory markers were extensively studied after exposing astrocytes to CSF to gain insights into their reactions. By analyzing the interactions between CSF and astrocytes, this study aims to shed light on the role of astrocytes in MS pathogenesis.

## Method

### Subjects

A total 16 subjects (**Table 1**) who visited Brain and Neurosurgery clinic at King Abdullah University hospital and Jordan University hospital and subjected to cerebrospinal fluid withdraw. MS patients should fulfil requirements for the diagnosis of definite MS using the 2017 modified McDonald criteria [20]. Patients had relapsing multiple sclerosis, diagnosed within a month of the presenting attack. CSF samples were collected before initiating treatment with disease modifying agents. They had no residual disability after the presenting attack remitted. Samples collection was started at 2nd of Feb, 2021 and extended to the 3rd of April, 2022. The Institutional Review Board of Jordan University of Science and Technology gave its approval to the study protocol (IRB# 136/136/2020). A formal informed consent was signed by each patient and control subject. The ethical guidelines of the institutional and/or national research committees, the 1964 Helsinki Declaration and its later amendments, or comparable ethical standards were followed in all procedures carried out in studies involving human participants.

**Table 1:** Demographic parameters of the study population.

Subject groups	No. of subjects	Female/male %	Age range	Average age ± SD
Control subjects	9	50%	21–70 years old	48.4±10.0
MS subjects	7	33.3%	23–56 years old	38.33±11.3

### CSF samples preparation

Collected samples of both healthy donors and MS diagnosed patients were centrifuged immediately after collection at 1000 xg for 10 mints at 4°C to remove any cellular debris. All samples were completely free from red blood cells. Clean supernatants were aliquoted and stored in aliquots at -80°C until being used.

### Cell Culture and maintenance

Gibco Human primary astrocyte glial cells were purchased from Fisher scientific (ThermoFisher Scientific, USA). Cells were maintained in pre-coated cell culture vessels with Geltrex matrix (ThermoFisher Scientific, USA), and they were supplemented with a complete medium of Dulbecco’s Modified Eagle Medium, 1X of N-2 supplement, 10% Fetal bovine serum, 20 ng/ml of Epidermal growth factor, 1% penicillin streptomycin (Euroclone), 1% HEPES (Euroclone) and 1% L-Glutamine (Euroclone). Cells were kept in a humidified incubator at 37°C with 5% CO2 until being ready for harvesting.

### Cell harvest and treatment with CSF

After being confluent, cells were collected and seeded in a Geltrex matrix pre-coated 12-well plates with a density of 150×103 and allowed to attach overnight. The day after, cells were starved for fetal bovine serum for 6 hours, then starvation medium was replaced with a fresh complete medium and cells were treated with CSF from both MS patients and healthy donors with a ration 1:1 and incubated for 72 hours. Control wells were running alongside the experiment and subjected to complete medium only. At the end of the incubation, conditioned media was collected and kept in -80°C until being used. Control and treated cells were harvested for subsequent RNA extraction.

### RNA extraction and qPCR gene expression assay

RNA was extracted from control and treated cells, following the manufacturer’s protocol (RNeasy® Plus Mini Kit, Qiagen). The amount of 500 ng was converted to cDNA following the protocol of PrimeScript RT Master Mix (Takara Bio Inc, Japan). RT-PCR technique was used to study the changes of expression of several genes affected by the treatment of astrocytes with MS and normal CSF, such as GFAP, GDNF, BDNF, VEGF, TNF-α, IL-1β, IL-6, and IL-10. Sequences of primers of each studied gene are listed in (**Table 2)**. Using PowerTrack™ SYBR Green Master Mix (Applied Biosystems, USA), RT-PCR mixes were prepared and allowed to run under conditions specified by the manufacturers protocol. ΔΔCT values for each treatment/each gene were calculated and further analyzed using GraphPad Prism statistical analysis software.

**Table 2:** List of forward and reverse primers used in qPCR experiment.

Gene name	Forward primers	Left primers
GFAP	TGTGAGGCAGAAGCTCCAGG	CCAGGGTGGCTTCATCTGCT
GDNF	TGAAACCAAGGAGGAACTGATTTT	GTCACTCACCAGCCTTCTATTTCTG
BDNF	CATCCGAGGACAAGGTGGCTTG	GCCGAACTTTCTGGTCCTCATC
VEGF	TGCAGATTATGCGGATCAAACC	TGCATTCACATTTGTTGTGCTGTAG
TNF- α	TAGCCCATGTTGTAGCAAACCC	TATCTCTCAGCTCCACGCCA
IL-1β	GCTCGCCAGTGAAATGATGG	GTCCTGGAAGGAGCACTTCAT
IL-6	AAAGCAGCAAAGAGGCACTGG	TTTCACCAGGCAAGTCTCCTCA
IL-10	CGCTGTGATCGATTTCTTCCC	AGATGCCTTTCTCTTGGAGCTTA
GAPDH	TCGGAGTCAACGGATTTGGT	TGAAGGGGTCATTGATGGCA

### Determination of IL-1β, IL-6, VEGF and BDNF concentrations via ELISA

The amount of IL-1β, IL-6, VEGF and BDNF secreted by astrocytes after the treatment with MS and healthy CSF were determined to confirm our qPCR results. Conditioned media of the experiment mentioned above was used for this purpose. Human IL-1β/IL-1F2 DuoSet, Human VEGF DuoSet, Human Total BDNF Quantikine ELISA kits (R&D Systems, USA) and Human Interleukin-6 ELISA Kit (SUNLONGBIOTECH, China) were used for this purpose, following their manufacturer’s protocol. Optical density was measured at 450 nm with correction at 570 nm. Concentrations were calculated using standard curve equations for each set and then analyzed using GraphPad Prism statistical analysis software.

### Statistical analysis

Excel was used to arrange data and calculate the ΔΔCT for gene expression studies. Further statistical analysis was performed using GraphPad Prism 8 software (GraphPad Software, Inc.). Results of relative gene expression and ELISA were statistically analyzed using *one-way ANOVA followed by Bonferroni’s post hoc test*, where statistically significant results were considered for p-value less than 0.05. Results are represented as mean±SEM.

## Results

### qPCR for CSF treated astrocytes

**Fig 1:**
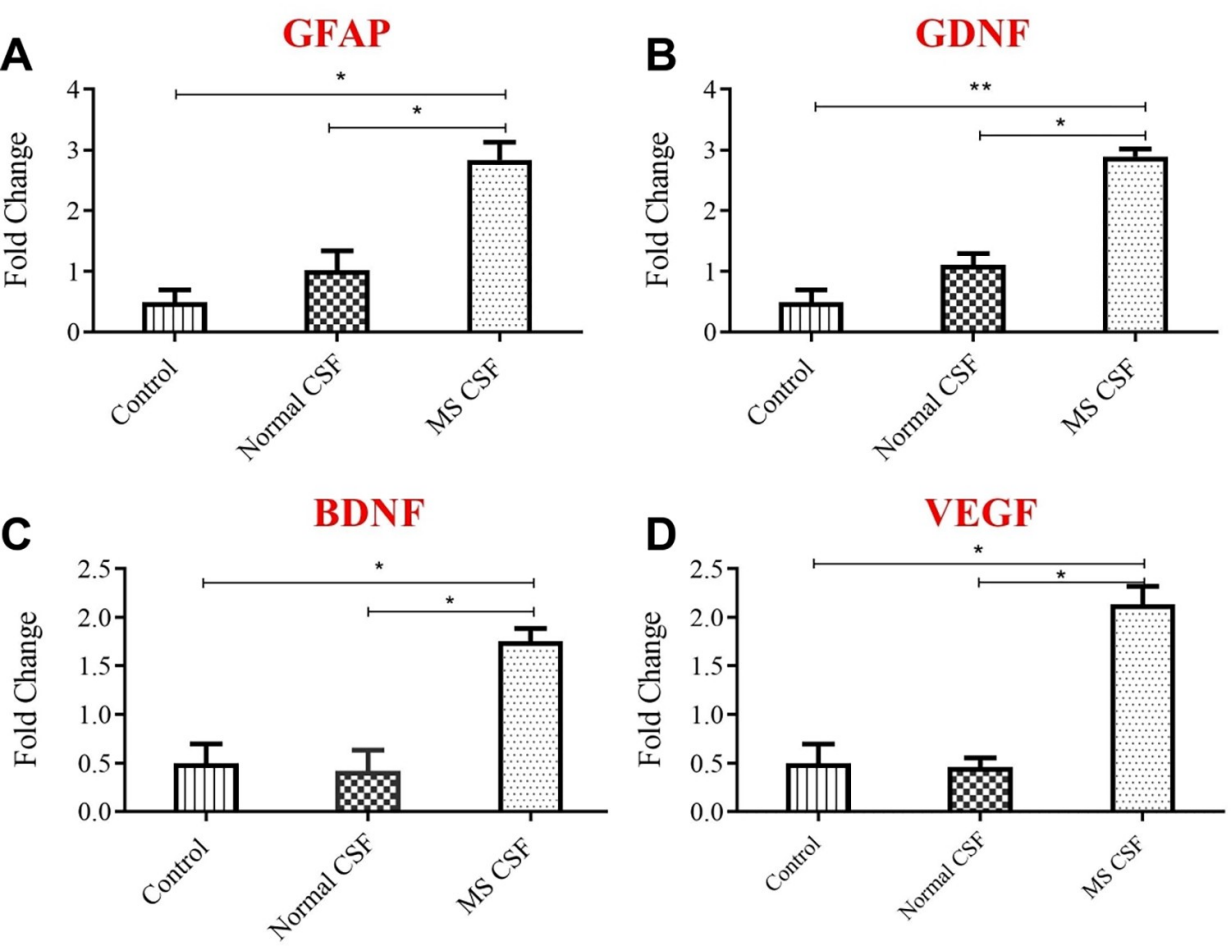
Changes of gene expression of neurotrophic factors using qPCR. Astrocyte primary cell culture treated with 50% concentrations of both MS-CSF (n = 7) and Ctrl-CSF (n = 9) for 72 hrs. Control groups were only exposed to culture medial only. The quantitative expression of mRNA was evaluated to several neurotrophic factors; (A) GFAP (B) GDNF (C) BDNF and (D) VEGF. Data were analyzed using *one-way ANOVA followed by Bonferroni’s post hoc test* and expressed as mean ± SEM. Level of significance was considered as * for p < 0.05, and ** for p < 0.01.

The effect of the secretory factors in the CSF of MS and healthy donors on the cultured astrocytes was evaluated by tracking the changes of expression of several genes, including GFAP, GDNF, BDNF, VEGF, TNF-α, IL-1β, and IL-6. Obviously, CSF derived from MS patients markedly and significantly elevated the expression of GFAP by 3 folds, compared to untreated cells. CSF derived from healthy donors slightly and insignificantly increased the expression of GFAP compared to untreated astrocytes. The amount of GFAP mRNA in MS-CSF treated cells is significantly higher than the amount found in Normal-CSF treated cells (**Fig 1A**). GFAP is an activation marker for astrocyte glial cells and the results indicate the presence of astrocyte activating factors in the central nervous system of MS suffering patients, which could be a survival and repair signal, as a response of central nervous system components of the disease.

Accordingly, the expression of two growth and survival signals, GDNF and BDNF, were elevated upon the exposure of astrocytes glial cells to CSF from MS patients. GDNF expression was markedly and significantly elevated about 3 folds by treatment with MS-CSF, compared to the other two groups (**Fig 1B**). Normal CSF showed a baseline stimulation of GDNF expression in treated astrocytes, which is higher but still insignificant compared to control group. BDNF expression in astrocytes was also affected and significantly increased after the treatment with MS-CSF but with a lesser extent compared to GDNF changes (**Fig 1C**). Moreover, normal-CSF did not affect BDNF mRNA amount, and its effect was like the control untreated group. Altogether, our results clarify the existence of several factors in the CSF of MS patients, which in turn activate astrocytes and stimulate their protective role in the CNS, through the release of many survival and growth factors. These data suggest the important role that astrocytes play in the pathogenesis of MS disease and sets astrocyte glial cells as a future therapeutic target of this disease. Companion to astrocyte glial cells activation via CSF of MS patients, mRNA of the migration factor, vascular endothelial growth factor VEGF, was significantly increased, while its level kept unaffected after the treatment of CSF of healthy donors (**Fig 1D**).

**Fig 2:**
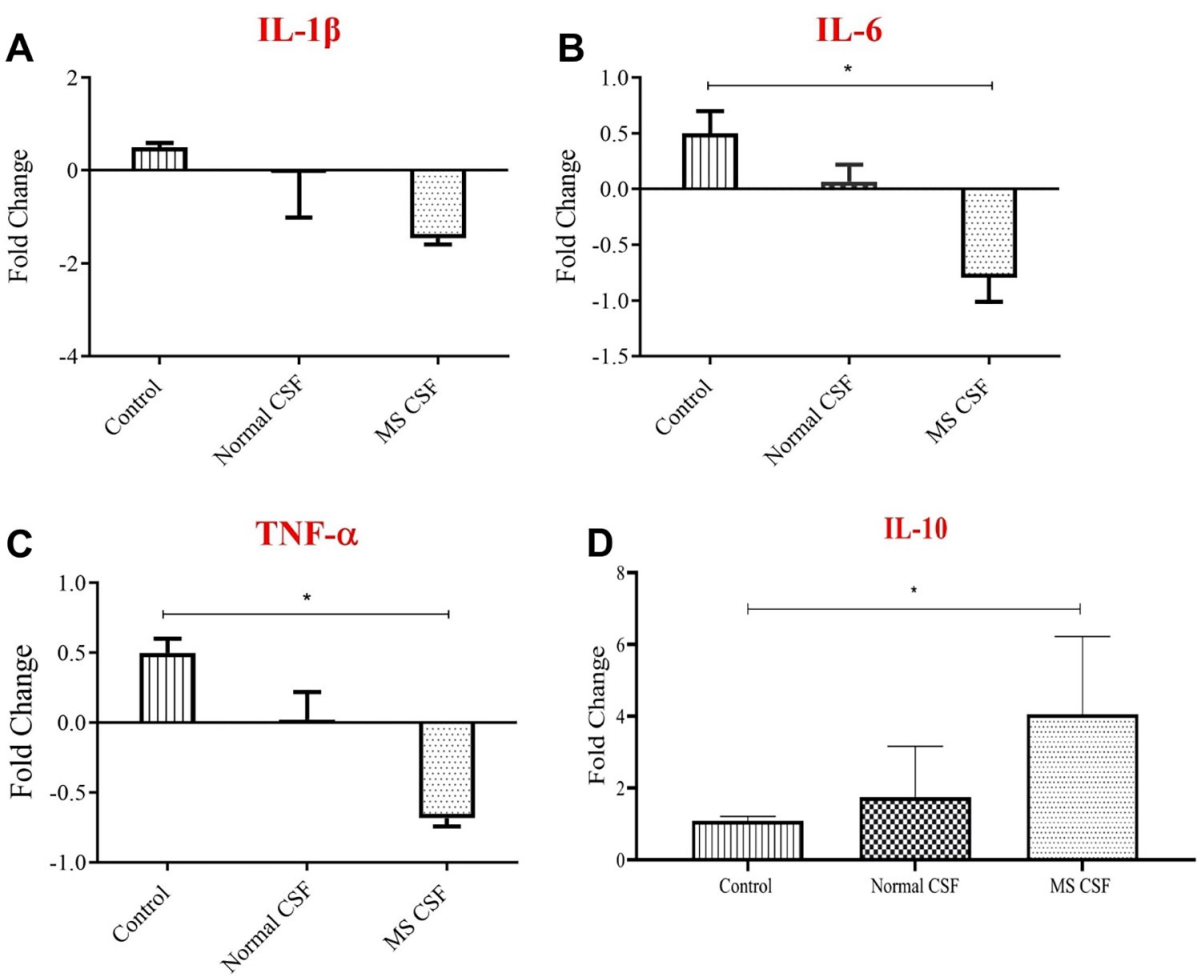
Changes of gene expression of inflammatory cytokines using qPCR. Astrocyte primary cell culture treated with 50% concentrations of both MS-CSF (n = 7) and Ctrl-CSF (n = 9) for 72 hrs. Control groups were only exposed to culture medial only. The quantitative expression of mRNA was measured for: (A) Interleukin-1β (B) Interleukin-6 (C) Tumor necrosis factor-α and (D) Interleukin-10. Data were analyzed using *one-way ANOVA followed by Bonferroni’s post hoc test* and expressed as mean ± SEM. Level of significance was considered as * for p < 0.05.

Interestingly, inflammatory responses related to astrocyte glial cells were also changed and they showed variant patterns by CSF from both MS and healthy donors. Three different cytokines, IL-1β, TNF-α and IL-6, were tested for the changes of their expression in astrocytes. Accordingly, control untreated astrocytes showed a baseline expression of the three types similarly, and MS-CSF was found to downregulate the expression of the three cytokine types in the tested astrocytes. However, this downregulation is shown to be insignificant for IL-1β (**Fig 2A**), in contrast to the other tested types, IL-6 and TNF-α (**Fig 2B and 2C**). The modest effect was observed for IL-6, which expression was changed from 0.5 folds observed in control group to -0.7 for MS-CSF treated group. Changes in the amount of mRNA of TNF-α is highly similar to that of IL-6, but with a slightly lesser extent. CSF isolated from healthy donors also downregulated the expression of these cytokines compared to their expression in the control untreated group, but these changes were not significant and considered negligible. On the other hand, the mRNA expression levels for anti-inflammatory cytokine IL-10 were detected in the astrocyte cells upon exposure to CSF from both MS patients and healthy donor. Interestingly, IL-10 mRNA expression level in astrocytes was affected and significantly increased 4.05 folds after the treatment with MS-CSF compared to untreated astrocyte. In addition, the IL-10 mRNA expression levels increased 1.74 folds in astrocyte upon treatment with CSF from healthy donor. Notably, the increase in IL-10 mRNA expression levels in CSF-treated astrocyte is significant compared to those cells that treated with CSF from healthy donor (**Fig 2D**).

These data support the idea that astrocytes play a role in the onset of MS-disease and suggested to have a major role in controlling it by reducing inflammatory responses and through the secretion of many growth and survival factors, which all together minimize the detrimental symptoms of the disease and set astrocytes as a future therapeutic target of multiple sclerosis.

### Amount of IL-1β, IL-6, VEGF and BDNF secreted by astrocytes

**Fig 3:**
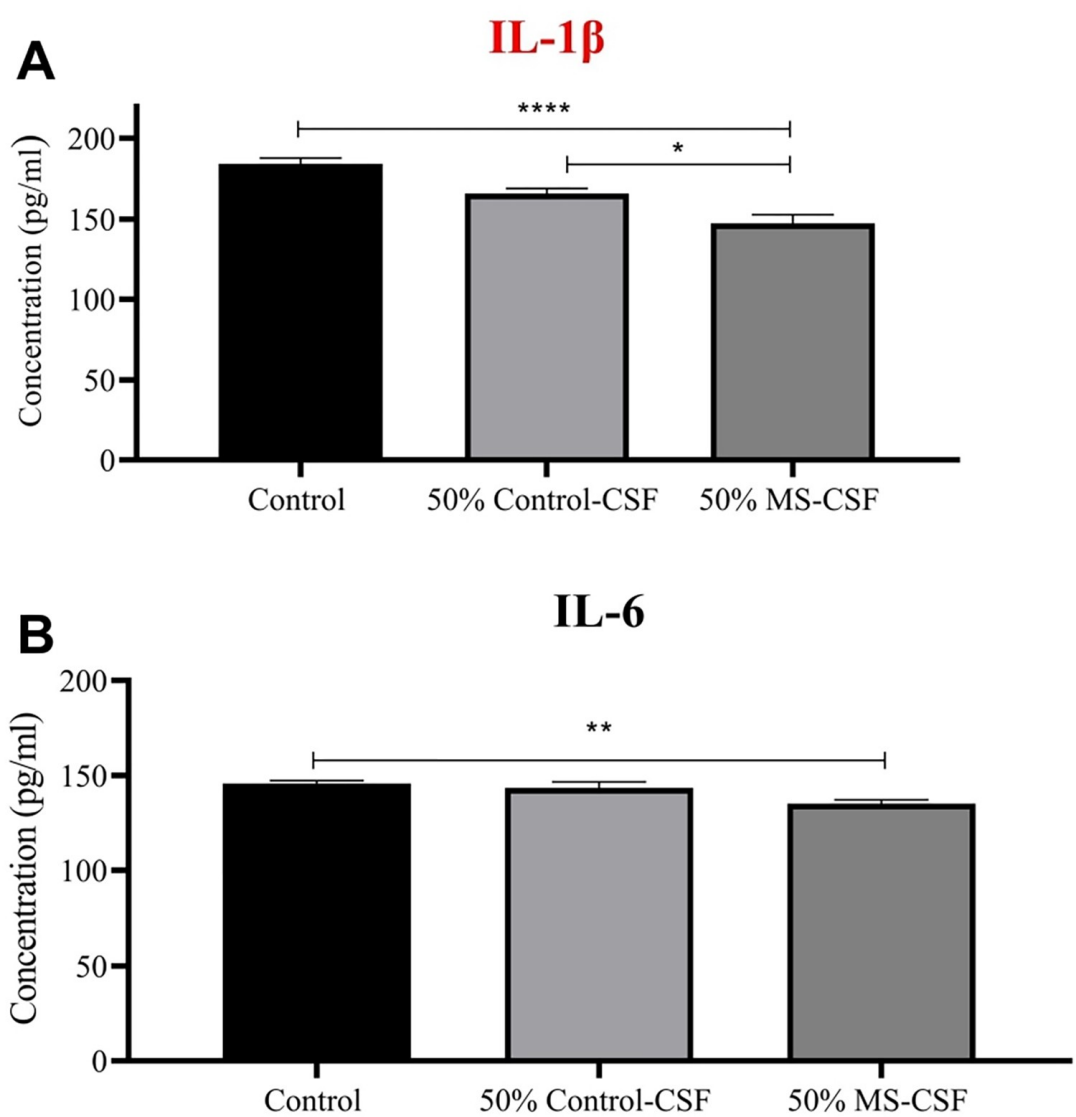
Enzyme linked immunosorbent assay results show the release of IL-1β and IL-6 inflammatory cytokines in cultured media upon treatment of Astrocyte primary cells with both MS-CSF (n = 7) and Ctrl-CSF (n = 9) for 72 hrs. Control cells kept untreated and only cultured with complete media. (A) 50% MS-CSF treatment significantly reduced the amount of secreted IL-1β compared to both Control untreated and Ctrl-CSF treated groups. In contrast, (B) IL-6 level was significantly reduced in MS-CSF treated group, where it was not affected by Ctrl-CSF treatment, compared to control untreated group. Data were analyzed using *one-way ANOVA followed by Bonferroni’s post hoc test* and expressed as mean ± SEM. Level of significance was considered as * for p < 0.05, **p < 0.01, ***P<0.0025, ****P<0.0001.

Enzyme linked immunosorbent assay was done to precisely count the amount of IL-β, IL-6, VEGF, and BDNF released by astroglial cells upon their exposure to CSF derived from both healthy and MS diagnosed donors. The concentration of IL-1β cytokine secreted to the conditioned media upon exposure of astrocytes to MS-CSF is significantly reduced when compared to control and Ctrl-CSF treated groups (**Fig 3A**). Similarly, IL-6 cytokine secretion to the conditioned media was also reduced significantly when astrocytes were exposed to MS-CSF. The change of IL-6 secretion to the conditioned media is said to be negligible when astrocytes get exposed to Ctrl-CSF when compared to the control group. These results ultimately confirm the data obtained by gene expression tests **(Fig 3B).**

**Fig 4:**
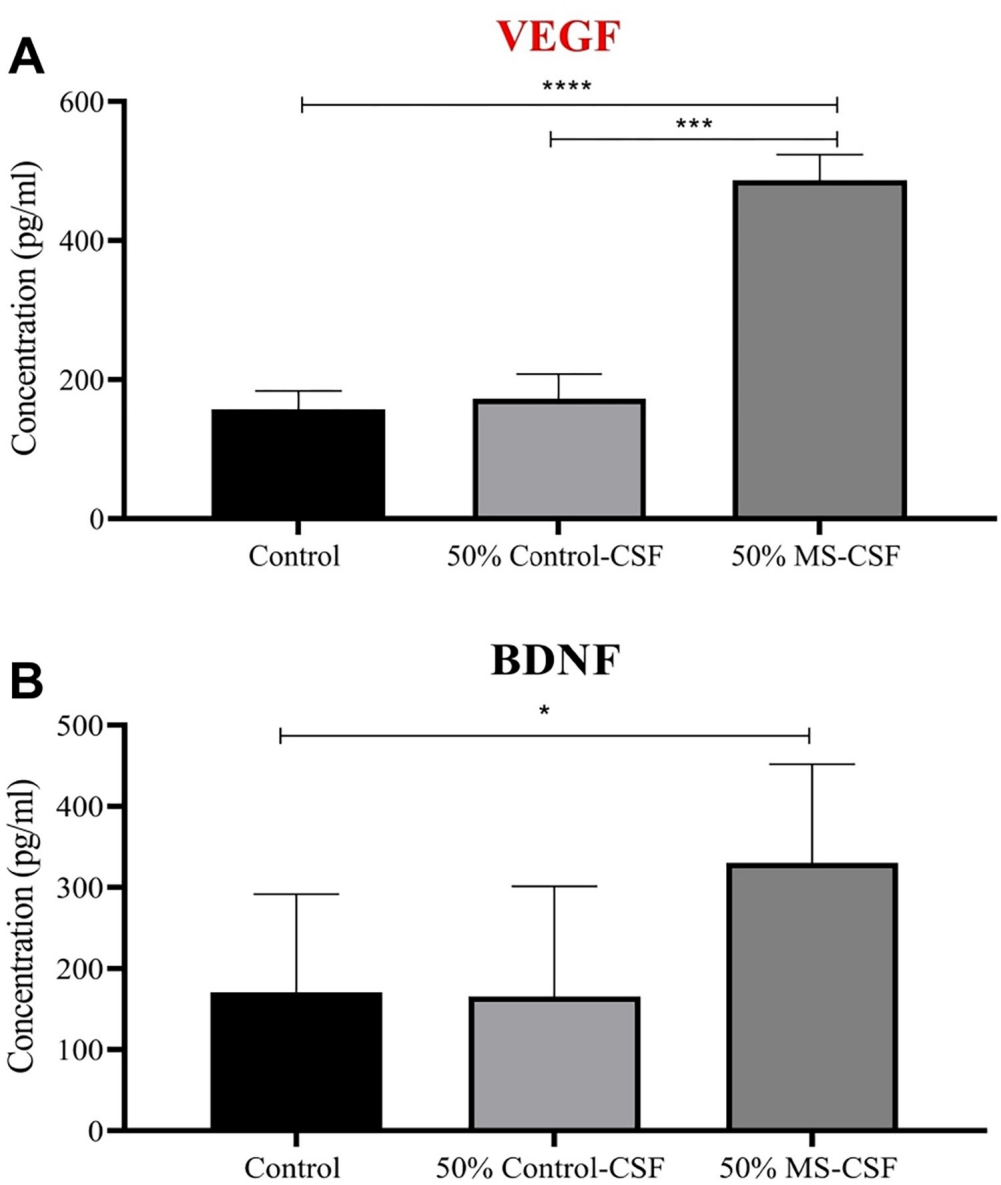
Enzyme linked immunosorbent assay results show the release of VEGF and BDNF neurotrophic factors in cultured media upon treatment of Astrocyte primary cells with both MS-CSF (n = 7) and Ctrl-CSF (n = 9) for 72 hrs. Control cells kept untreated and only cultured with complete media. (A) 50% MS-CSF treatment significantly increased the amount of secreted VEGF compared to both Control untreated and Ctrl-CSF treated groups. In contrast, (B) BDNF level was significantly elevated in MS-CSF treated group, where it was not affected by Ctrl-CSF treatment, compared to control untreated group. Data were analyzed using *one-way ANOVA followed by Bonferroni’s post hoc test* and expressed as mean ± SEM. Level of significance was considered as * for p < 0.05, **p < 0.01, ***P<0.0025, ****P<0.0001.

The elevated VEGF expression observed in qPCR was further confirmed here. As seen in (**Fig 4A**) VEGF amount secreted by MS-CSF treated astrocytes is significantly higher by nearly three folds compared to both control and Ctrl-CSF treated groups. Obviously, VEGF level was not changed significantly in Ctrl-CSF treated group when compared to the control group. In a similar pattern, the highest amount of secreted BDNF was observed in the group that was exposed to MS-CSF. This increase was significant compared to control treatment group. On the other hand, Ctrl-CSF did not show any effect on BDNF secretion by the exposed astrocytes (**Fig 4B**).

## Discussion

Despite increasing interest in the association of astrocytes with neurodegenerative diseases, the specific effects of MS-CSF on astrocytes had not been previously investigated. However, To our knowledge, the effect of CSF from MS patients on astrocytes has not been reported. As astrocytes are important in the CNS immune response and can relay inflammation, we examined gene expression of MS-associated cytokines in CSF-treated astrocytes. TNF-α, IL-1β and IL-6 have been measured for their involvement in the immunopathology of MS. Moreover, the change in expression levels of GFAP as a marker for astrocytic activation was evaluated. As BDNF is described as a neuronal-survival gene and the link between BDNF and MS has been poorly investigated, we also aimed to investigate the expression analysis of BDNF. GDNF has also been reported to have the ability of promoting axonal regeneration and remyelination of axons, therefore, in the current study, we assess whether GDNF expression was changed in CSF-treated astrocyte or not.

As MS is a prototypical neuroinflammatory disease, we used CSF obtained from both MS and healthy patients to investigate the role of activated astrocytes in this pathology. In MS, immune cells enter the CNS parenchyma from the periphery, damaging the myelin sheath and axon. There is increasing evidence that glial cells contribute to MS pathology, especially astrocytes [21]. The role of glial cells, specially, astrocytes in MS has been studied commonly in mice through the EAE model, not in human MS patients and applying of this data from rodent to human might not necessarily be accepted [22]. In this study, several interesting findings emphasize the neuroprotective role of astrocyte upon their treatment with MS-CSF. Our data show that astrocytes may have the ability to protect neuronal population and can efficiently suppress the cascade of inflammatory responses associated with the phenotype of MS disease.

Recent studies have reported that the morphology of astrocytes is complex and region-dependent, suggesting heterogeneity in their structure, distribution and function [23,24]. Astrocytes are widely involved in CNS development and function. In addition, previous studies have focused on the idea that astrocytes are dynamic cells that express diverse receptors, allowing them to respond to a wide variety of neurotransmitters, including neuropeptides, growth factors, cytokines and toxins [25].

As mentioned above, besides the astrocytes’ contribution to CNS function and homeostasis, their role under pathological circumstances, particularly in MS, was studied. Consequently, there is an emerging evidence that the astrocytes respond to insult by forming a glial scar, but play important role in disease mechanism and pathogenesis [26]. EAE model studies have revealed the protective and detrimental roles of this complex cell population in mediating inflammation, tissue damage, and repair. The findings of this study demonstrated a significant reduction in the expression levels of TNF-α, IL-1β, and IL-6 in MS-CSF treated astrocytes compared to healthy CSF treated and untreated groups. In addition, the data of our study revealed a significant increase in mRNA expression levels of anti-inflammatory cytokine IL-10 in MS-CSF treated cells compared to untreated and healthy-CSF treated groups. These results align with previous studies indicating that astrocytes can display both pro-inflammatory and anti-inflammatory effects, depending on the nature and severity of the disease [27,28]. Accordingly, reactive astrocytes possibly release diverse molecules and influence adjacent cells, can exhibit pro-inflammatory effects and exaggerate the neurological damage, or display anti-inflammatory effects, promoting protection and repair. In this context, astrocytes can be characterized as "A1" or "A2," [29], analogous to the categories used for macrophages “M1” and “M2”, with A1 astrocytes being detrimental [30], and A2 astrocytes being anti-inflammatory and neuroprotective [31].

In this study, we also evaluated various neurotrophic factors, including BDNF and GDNF, and their roles in MS. BDNF and GDNF are small proteins that mediate trophic events in neurons and play important roles in CNS development and maturation. In general, neurotrophic factors work by binding to cell surface receptors that signal neurons to survive. the study revealed a significant increase in BDNF expression in astrocytes treated with MS-CSF. BDNF was considered a neuroprotective factor involved in autoimmunity and inflammation and showed beneficial effects in animal models of MS [32]. Moreover, BDNF has been reported to display beneficial effects in EAE model [33]. This finding provides an experimental and clinical evidence that BDNF is a major element in neuro-inflammation modulation, neuroprotection and neuro-repair in MS disease, which makes the BDNF a promising candidate for new therapeutic strategies in MS. On the other hand, GDNF promotes the survival of many types of neurons such as dopaminergic and motor neurons. Also, it has an ability to promote axon regeneration and myelination after spinal cord injury [34]. Previous studies revealed that this factor can rescue dopaminergic neurons in animal models of Parkinson’s disease and prevent motor neuron degeneration in animal models of amyotrophic lateral sclerosis (ALS) [35,36]. Our data demonstrate that the expression of GDNF is increased in astrocytes upon treatment with CSF from multiple sclerosis patients. The up-regulation of GDNF in MS-CSF treated astrocytes suggested its role in neuronal survival, axon regeneration, and remyelination.

VEGF-A is a growth factor produced by Astro-glial cells, exerting a mitogenic effect on both astrocytes and oligodendrocyte progenitor cells (OPCs) [37]. The role of VEGF-A, as a one of the key factors involved in angiogenesis, in MS is controversial. Harandi and his colleagues reported that increase in the serum VEGF level may be involved in MS pathogenesis [38]. Therefore, it could be used as a prognostic and predictive biomarker for MS disease. In contrast, several cumulative studies indicated the anti-inflammatory protective and neurotrophic properties of VEGF in MS late phase onset of the disease [39,40]. This controversial because it is deemed an over-simplification of the heterogeneous complexity of astrocytes structures and functions. VEGF-A was known to stimulate neuron axonal growth and support injured sites in MS disease [37,41]. The effect of VEGF in MS disease extends beyond angiogenesis stimulation to supply injured sites with enough nutrients, it was found that VEGF-A stimulate the differentiation and migration of oligodendrocytes precursor cells into the demyelinated lesions [40,41]. Our results are consistent with the aforementioned studies, as we found that astrocytes stimulated with MS-CSF secreted a significant amount of VEGF-A, emphasizing their role in the improvement and reduction of the disease pathogenic phenotype.

As of right now, it is evident that astrocytes are crucial for attracting, guiding, and keeping leukocytes at lesion sites, which creates the positive-feedback inflammatory loop responsible for mediating the MS disease. Through their innate neurotoxic actions and the activation of other cells, astrocytes also cause tissue damage, which accelerates the course of illness and neurodegeneration. On the other hand, it is also known that astrocytes play important functions in promoting neuroprotection and repair as well as limiting harmful inflammation [42]. These combined effects highlight the significance of astrocytes as essential components of MS pathophysiology and their rapid and effective regulation [43]. However, the variety of roles suggests that astrocytes are passive observers who are influenced by a range of context-specific elements, including the area in which they reside, the kind and intensity of CNS insults, the local pro- or anti-inflammatory mediators, and interactions with resident and immune cells [44]. Interestingly, research on astrocyte biology is currently concentrating on the astrocytic subsets’ heterogeneity and characterization. While the diversity of astrocytes in terms of the factors they produce and the roles they display have been extensively studied, there is a great deal of research being done on their many regulatory and activation signals, plasticity, and interactions with adjacent cells.

Nevertheless, this study does have some limitations that must be addressed. First, number of samples in both groups healthy and MS patients was small due to some difficulties to obtain CSF samples from the patients. In addition, due to the small sample volume, we couldn’t perform all of the planned experiments and assays such as the determination of the CSF components. There is a need to identify the differences in the components of healthy and MS CSF. This study only dealt with limited number of cytokines and neurotrophic factors, and further expansion of the effect of CSF on different cytokines and factors need to be elucidated in the future.

In conclusion, the study established an in vitro model using human astrocytes to investigate their role in MS pathology. We used this model to investigate the profile of astrocytes after stimulation with MS-CSF and healthy-CSF which may be of a great value to understand the role of astrocytes in MS. The results indicated that astrocytes have neuroprotective effects and play a significant role in the onset of MS. The findings shed light on the complexities of astrocyte responses to MS-CSF and their potential as therapeutic targets for the disease.

## Supporting information

S1 File (DOCX)

S2 File (XLSX)
