# Supplementary material for: The neuroprotective effect of human primary astrocytes in multiple sclerosis: In vitro model
Source: PLoS One. 2024 Apr 2;19(4):e0300203. doi: 10.1371/journal.pone.0300203 (PMC10987000; doi:10.1371/journal.pone.0300203)
Supplement: S1 File — (DOCX) [file pone.0300203.s001.docx]

# Request for Change to Authorship

Check to confirm you have read [*PLOS ONE*’s authorship policy](http://journals.plos.org/plosone/s/authorship).

The authorship criteria for *PLOS ONE*, summarized below, are based on those outlined by the International Committee of Medical Journal Editors (ICMJE):

1. Conception and design of the work, acquisition of data, or analysis and interpretation of data
2. Drafting the article or revising it critically for important intellectual content
3. Final approval of the version to be published
4. Agreement to be accountable for all aspects of the work

Authors should meet all of the criteria; the contributions of all authors will be disclosed in the final publication. Any contributions that fall short of the criteria should be named in the Acknowledgments section of the manuscript. It is your responsibility to ensure that anyone named in the Acknowledgments consents to being named.

Check to confirm that all authors (including those to be added or removed) consent to the changes detailed below.

| **Reason for change in author list**  Please briefly describe the reason for adding/removing an author. | After our initial submission and receiving the comments of the reviewers, in which they suggest performing new extra experiments. The added author (Raneen Qasem) studied the anti-inflammatory cytokines (IL-10) and BDNF level in CSF samples. Which was crucial for the final version of our manuscript in terms of methodology, data collection, analysis and interpretation. |
| --- | --- |

# Final manuscript information

| **Manuscript number**  e.g., PONE-D-17-00000 | PONE-D-23-20017R2 |
| --- | --- |
| **Complete author list, in correct order**  Please note any equal contributors with asterisks (*) or hashes (#) | Amer Imraish, Tuqa Abu Thiab, Mohammad Alsalem, Saeed Dahbour, Hiba khleif, Basha'er Abu-Irmaileh, Raneen Qasem and Khalid El-Salem |
| [**Financial Disclosure**](http://journals.plos.org/plosone/s/disclosure-of-funding-sources) – including any additions/deletions necessary due to the change in authorship | We would like to thank the Deanship of Scientific Research in The University of Jordan for funding this project (grant number: 2352).  The funders had no role in study design, data collection and analysis, decision to publish, or preparation of the manuscript. |
| [**Competing Interests**](http://journals.plos.org/plosone/s/competing-interests) – including any additions/deletions necessary due to the change in authorship | The authors have declared that no competing interests exist. |
| [**Acknowledgments statement**](http://journals.plos.org/plosone/s/submission-guidelines#loc-acknowledgments)  Please acknowledge any removed authors if they contributed to the study in any way, as well as members of any author groups who do not meet our authorship criteria. | NA |

# Adding authors

## Individual author addition #1

| **Full name** | Raneen Qasem |
| --- | --- |
| **Email address** | raneenqasim1@gmail.com |
| **Full affiliation** | Department of Biological Sciences, School of Science, The University of Jordan, Amman 11942, Jordan |

| This person contributed to **all** of the following:   1. Conception and design of the work, acquisition of data, or analysis and interpretation of data 2. Drafting the article or revising it critically for important intellectual content 3. Final approval of the version to be published 4. Agreement to be accountable for all aspects of the work |  |
| --- | --- |
| **Specific contributions:** | |
| Conceptualization |  |
| Data Curation |  |
| Formal Analysis |  |
| Funding Acquisition |  |
| Investigation |  |
| Methodology |  |
| Project Administration |  |
| Resources |  |
| Software |  |
| Supervision |  |
| Validation |  |
| Visualization |  |
| Writing – Original Draft Preparation |  |
| Writing – Review & Editing |  |

## Individual author addition #2 (if applicable)

| **Full name** |  |
| --- | --- |
| **Email address** |  |
| **Full affiliation** |  |

| This person contributed to **all** of the following:   1. Conception and design of the work, acquisition of data, or analysis and interpretation of data 2. Drafting the article or revising it critically for important intellectual content 3. Final approval of the version to be published 4. Agreement to be accountable for all aspects of the work |  |
| --- | --- |
| **Specific contributions:** | |
| Conceptualization |  |
| Data Curation |  |
| Formal Analysis |  |
| Funding Acquisition |  |
| Investigation |  |
| Methodology |  |
| Project Administration |  |
| Resources |  |
| Software |  |
| Supervision |  |
| Validation |  |
| Visualization |  |
| Writing – Original Draft Preparation |  |
| Writing – Review & Editing |  |

## Author group addition (if applicable)

| **Group or consortium name** |  |
| --- | --- |
| **Author who represents group** |  |

# Removing authors

## Author removal #1

| **Full name** |  |
| --- | --- |

| This person **did not** contribute to all of the following:   1. Conception and design of the work, acquisition of data, or analysis and interpretation of data 2. Drafting the article or revising it critically for important intellectual content 3. Final approval of the version to be published 4. Agreement to be accountable for all aspects of the work |  |
| --- | --- |
| This person consents to being acknowledged in the published paper. |  |

## Author removal #2 (if applicable)

| **Full name** |  |
| --- | --- |

| This person **did not** contribute to all of the following:   1. Conception and design of the work, acquisition of data, or analysis and interpretation of data 2. Drafting the article or revising it critically for important intellectual content 3. Final approval of the version to be published 4. Agreement to be accountable for all aspects of the work |  |
| --- | --- |
| This person consents to being acknowledged in the published paper. |  |
